# NeuroSCORE is a genome-wide omics-based model that identifies candidate disease genes of the central nervous system

**DOI:** 10.1038/s41598-022-08938-y

**Published:** 2022-03-31

**Authors:** Kyle W. Davis, Colleen G. Bilancia, Megan Martin, Rena Vanzo, Megan Rimmasch, Yolanda Hom, Mohammed Uddin, Moises A. Serrano

**Affiliations:** 1 Bionano Genomics, Lineagen Division, Inc., 9540 Towne Center, Dr. #100, San Diego, CA 92121 USA; 2 College of Medicine, Mohammed Bin Rashid University of Medicine and Health Sciences, Dubai, UAE; 3 Cellular Intelligence (Ci) Lab, GenomeArc Inc., Toronto, ON Canada

**Keywords:** Disease genetics, Neurodevelopmental disorders, Medical genomics

## Abstract

To identify candidate disease genes of central nervous system (CNS) phenotypes, we created the Neurogenetic Systematic Correlation of Omics-Related Evidence (NeuroSCORE). We identified five genome-wide metrics highly associated with CNS phenotypes to score 19,601 protein-coding genes. Genes scored one point per metric (range: 0–5), identifying 8298 scored genes (scores ≥ 1) and 1601 “high scoring” genes (scores ≥ 3). Using logistic regression, we determined the odds ratio that genes with a NeuroSCORE from 1 to 5 would be associated with known CNS-related phenotypes compared to genes that scored zero. We tested NeuroSCORE using microarray copy number variants (CNVs) in case–control cohorts and aggregate mouse model data. High scoring genes are associated with CNS phenotypes (OR = 5.5, *p* < 2e^-16^), enriched in case CNVs, and mouse ortholog genes that cause behavioral and nervous system abnormalities. We identified 1058 high scoring genes with no disease association in OMIM. Transforming the logistic regression results indicates high scoring genes have an 84–92% chance of being associated with a CNS phenotype. Top scoring genes include *GRIA1, MAP4K4, SF1, TNPO2,* and *ZSWIM8*. Finally, we interrogated CNVs in the Clinical Genome Resource, finding the majority of clinically significant CNVs contain high scoring genes. These findings can direct future research and improve molecular diagnostics.

## Introduction

A cholera epidemic swept across the globe in 1819 from India through the Middle East, Europe, and to America. British- and American-based physicians John Snow and Amariah Brigham both studied cholera and produced maps of the deaths in New York and London—Brigham’s in 1831 and Snow’s in 1855^1,2^. Both maps used different overlapping evidence, such as trade routes and drinking water systems, to illustrate a confluence of variables leading to new insights about cholera and, ultimately, public health remedies. Today, geneticists can take a similar approach, with different types of maps, to identify the genetic mechanism of diseases. With genome-wide, multi-omic analyses we can now overlay these datasets on the human genome and correlate these with phenotypes of the central nervous system (CNS) to identify candidate disease genes thereby improving research into candidate disease genes, diagnostics and, eventually, therapies.

Identifying disease or risk genes for conditions of the CNS has been a slow process, with current diagnostic rates for children with a broad range of neurological or developmental conditions ranging from 31% to 53%^3,4^ (undergoing multiple clinical tests) and approximately 32% in adults^5^. Diagnostic rates using whole exome sequencing and chromosomal microarray vary within particular phenotypes, ranging from approximately 16% in autism spectrum disorder (ASD)^6^, 23% in corpus callosum anomalies^7^, and 42% in early-onset epileptic encephalopathies^8^. These conditions all likely have substantial unrecognized genetic contribution and a recent study of developmental disorders found that more than 1000 additional genes are expected to contribute, either alone or in combination, to neurodevelopmental disorders^9^.

Identifying CNS-disease genes is complicated, as genetic CNS diseases are caused by multiple pathogenic mechanisms^4^, display multiple forms of inheritance, are characterized by allelic heterogeneity, reduced penetrance, pre/perinatal lethality, and variable expressivity, are difficult to study in vivo, have phenotypes that exist on a spectrum (e.g. ASD), have variable age of onset, have broad descriptions (e.g. “developmental delay”), and many genes characterized in the 1980s–1990s have received disproportionate study leading to many unstudied genes, colloquially called the “ignorome”^10^.

Previous attempts to identify candidate disease genes have used multiple approaches, including statistical modeling for probability of causing a haploinsufficiency-related condition (pLI score^11^) or identifying genes with regional coding constraint^12^. Other systems rely on direct counts of particular type of variant observed in a control population (gnomAD’s observed/expected metrics^13^). Lastly, systems have been devised to search for disease-specific genes, such as ForecASD^14^ and candidate ASD genes, or used specific data such as gene expression patterns in the brain-coX model^15^. These systems, while useful, are limited and no multi-omic system has yet been devised for CNS phenotypes.

**Figure 1:**
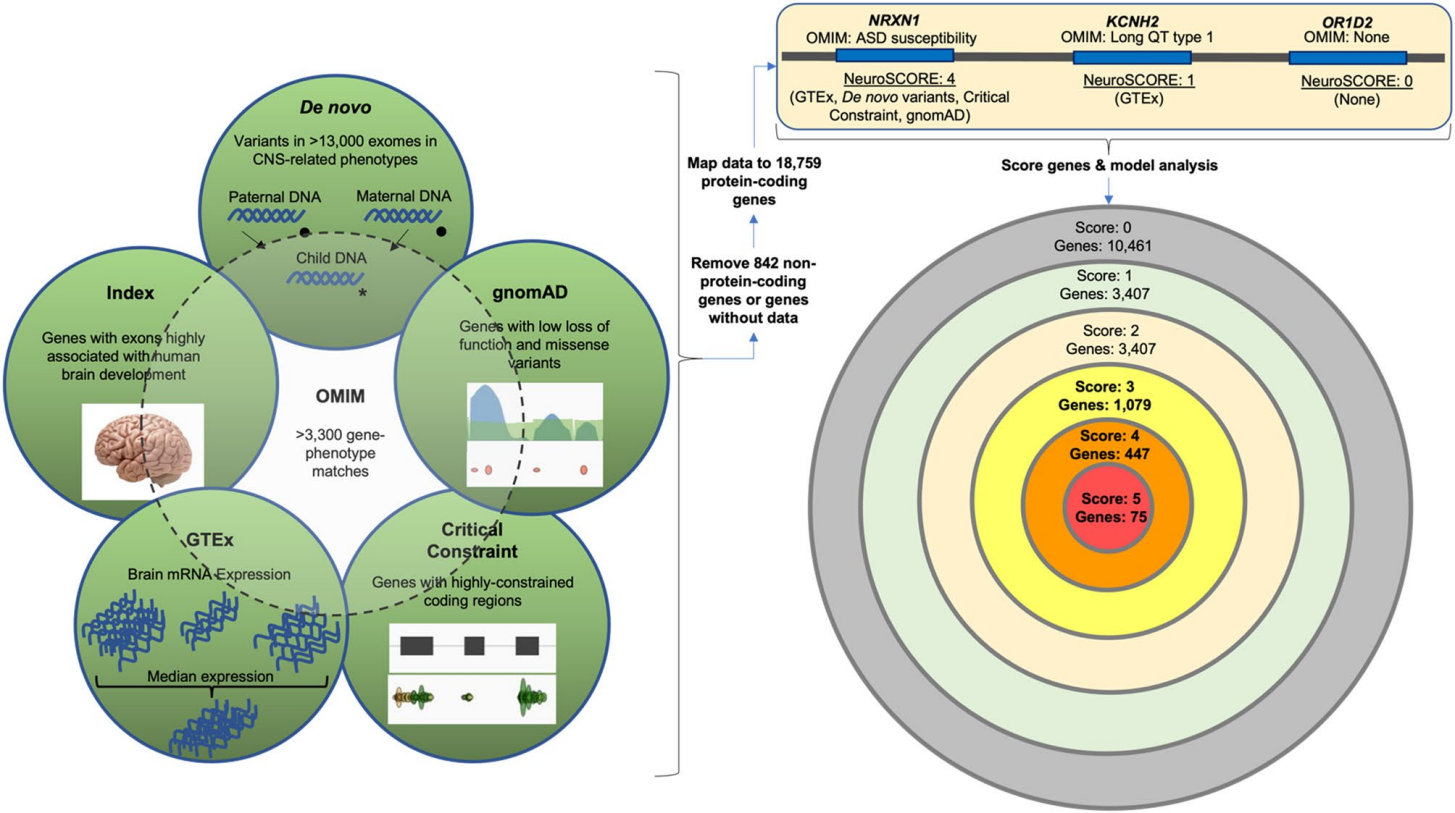
Schematic of final NeuroSCORE model. Key: De novo: De Novo Database; gnomAD: Genome Aggregation Database; Critical Constraint: Critically constrained coding regions database; GTEx: Gene-Tissue Expression database; Index: Database of genes based on Uddin et al.^66^; OMIM: Online Mendelian Inheritance in Man database (see “Methods”).

As a diagnostic laboratory focused on neurological and developmental phenotypes, we sought to create a model that identified and prioritized candidate disease genes. We began with two foundational concepts, the first being the use of a multi-omics approach to account for different potential disease mechanisms and characteristics of genes that underlie known neurogenetic conditions. The second concept is developmental brain dysfunction, which posits that distinct clinical diagnoses affecting the cognitive, motor, neuropsychiatric, neurobehavioral, or neuroanatomical domains can be classified into an overarching umbrella term that describes the underlying cause of clinical symptoms because they share similar underlying genetic risk factors^16,17^. By leveraging large scale -omics databases supporting these two foundational concepts with a clinical database, we created NeuroSCORE: the Neurogenic Systematic Correlation of Omics-Related Evidence (Fig. 1). We believe NeuroSCORE is the first multi-omic model to assess most protein-coding genes and focused broadly on CNS phenotypes.

## Results

### Constructing and assessing the NeuroSCORE model

To build a comprehensive and clinically useful model, we chose genome-wide databases with gene-specific data to assess expression, constraint, and other properties of protein coding genes (dependent variables; see “Methods”). Using Pearson’s chi squared, we determined if any of the identified nine gene metrics were correlated with our outcome measure of genes currently associated with a condition that has one or more CNS-related phenotypes in the OMIM database. Briefly, these gene metrics identify genes with: significant enrichment of de novo variants in individuals affected with neurological or neurodevelopmental conditions (“de novo” metric), one or more exons with high expression throughout human brain development and with low mutation burdens (“Index” metric), one or more regions of coding constraint at the 95th or 99th percentiles as measured in samples from the Genome Aggregation Consortium (gnomAD) called “critically constrained regions” (“CCR 99” and “CCR 95” metrics), median brain expression at or above 10 transcripts per million as measured across all brain region samples from the Genotype-Tissue Expression (GTEx) database (GTEx metric), low levels of loss-of-function or missense variants in gnomAD (“gnomAD LOF” and “gnomAD MIS” metrics), and enrichment in copy number variants (CNVs) in a neurodevelopmentally affected cohort at different levels of statistical significance (“Coe 1” and “Coe 2” metrics).

Eight of these nine variables were significantly associated with currently known CNS-related disease genes (Table 1), including the Genotype-Tissue Expression (GTEx) database genes, de novo genes, genes with regions of critical constraint at both the 99th and 95th percentiles, gnomAD LOF and MIS genes, Index genes, and Coe 1 genes metrics (all *p* < 0.05). The Coe 2 gene metric was not correlated (*p* = 0.10), and data were excluded. Using the remaining eight significant metrics, we then constructed a multiple logistic regression model. Main effects from multiple logistic regression modeling indicated that five of the eight metrics were significantly and positively associated with odds ratios (ORs) above 1.0 for the outcome measure of CNS-related disease genes.

**Table 1:** NeuroSCORE gene metric association of genes with CNS-related clinical features.

Gene metric	Pearson’s χ^2^		Logistic regression			Wald test		Total genes
	χ^2^	*p*	OR	95% CI	*p*	*F*	*p*	
**De novo**	**67.0**	** < 1e**^**-4**^	**2.2**	**1.7**–**3.0**	** < 1e**^**-4**^	**27.5**	** < 1e**^**-4**^	**487**
**Index**	**171.1**	** < 1e**^**-4**^	**1.9**	**1.5**–**2.3**	** < 1e**^**-4**^	**39.4**	** < 1e**^**-4**^	**4636**
**CCR 99**	**82.9**	** < 1e**^**-4**^	**1.8**	**1.4**–**2.3**	** < 1e**^**-4**^	**24.3**	** < 1e**^**-4**^	**1444**
**GTEx**	**154.0**	** < 1e**^**-4**^	**1.7**	**1.4**–**2.0**	** < 1e**^**-4**^	**30.7**	** < 1e**^**-4**^	**6069**
**gnomAD LOF**	**55.5**	** < 1e**^**-4**^	**1.4**	**1.1**–**1.6**	**5e**^**-4**^	**12.1**	**5e**^**-4**^	**2896**
CCR 95	19.8	< 1e^-4^	0.9	0.8–1.1	0.4	NA	NA	7049
gnomAD MIS	20.2	< 1e^-4^	1.8	0.8–4.5	0.2	NA	NA	144
Coe 1	4.2	.04	1.2	0.9–1.4	.10	NA	NA	3116
Coe 2	2.7	.10	NA	NA	NA	NA	NA	3732

Bold text indicates variables used in the final model; OR: odds ratio; 95% CI 95% confidence interval; CCR 95 or 99: genes with ≥ 1 critically constrained coding region at the 95th or 99th percentiles; GTEx: gene-tissue expression database; gnomAD LOF and MIS: gnomAD genes with upper bound of loss-of-function or missense observed/expected metric < 0.35; NA: not applicable.

These five variables became our final NeuroSCORE model: de novo genes, Index genes, critically constrained genes at the 99th percentile, GTEx genes, and gnomAD LOF genes. Two-way interactions were not assessed; however, all ORs are within a similar range suggesting these variables have a similar strength and thus no single variable is having an outsized influence on the model.

**Figure 2:**
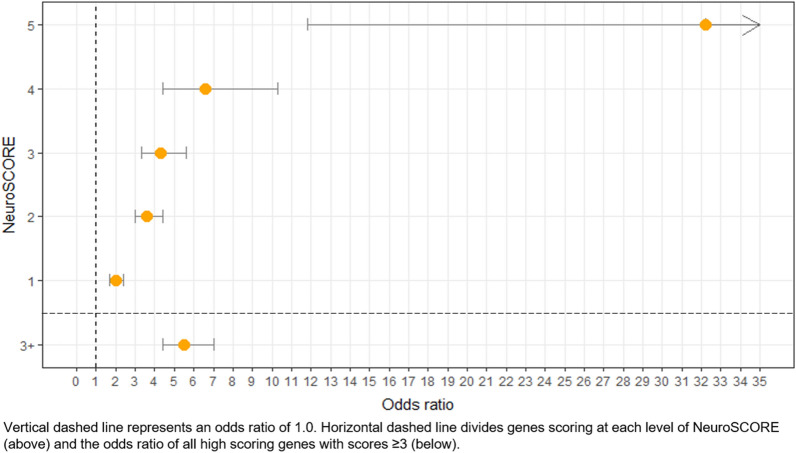
Odds ratio of genes associated with CNS-related phenotypes by NeuroSCORE.

NeuroSCORE creates different scoring levels for genes from 0 to 5 points. We then used logistic regression to investigate the relative enrichment of currently known CNS-related disease genes within each scoring level compared to genes that scored zero and calculated the OR for genes identified at each scoring level (Table 2 and Fig. 2). ORs increased with each increase in NeuroSCORE, ranging from 2.0 to 32.2 (*p* < 5e^-8^). Next, we calculated the OR for genes that met a majority of the NeuroSCORE metrics (≥ 3 points), which we considered to be “high scoring” genes (Table 2). As this set of high scoring genes is significantly associated with CNS-related disease genes, we focused the remaining analyses on the high scoring genes (*N* = 1601). As a negative control, we calculated the OR for genes that scored 0 in our model to be associated with non-CNS-related phenotypes. This set of genes was significantly more likely to be associated with genes causing non-CNS-related phenotypes (OR = 1.7, 95% CI 1.5–1.9, *p* < 2e^-16^), showing these genes are enriched for non-CNS-related conditions.

**Table 2:** NeuroSCORE model shows increasing odds ratios with increasing point totals.

NeuroSCORE	Logistic regression results			OMIM genes				
	OR	95% CI	*p*	Total genes	CNS phenotype	No CNS phenotype	No phenotype	Absent from OMIM
5 of 5	32.2	11.8–132.7	5.7e^-9^	75	58	3	14	2
4 of 5	6.6	4.4–10.3	2.0e^-16^	447	121	29	297	11
3 of 5	4.3	3.3–5.6	2.0e^-16^	1079	241	91	747	135
2 of 5	3.6	3.0–4.4	2.0e^-16^	3407	603	284	2520	644
1 of 5	2.0	1.7–2.4	1.1e^-14^	3290	489	409	2392	453
0 of 5	NA	NA	NA	10,461	715	1389	8357	2734
≥ 3 of 5	5.5	4.4–7.0	2.0e^-16^	1601	420	123	1058	148

OR: odds ratio; 95% CI 95% confidence interval; NA: not available; high scoring genes are genes identified by ≥ 3 gene sets; OMIM data current as of July 31st, 2021.

### High scoring genes and mouse model organism data

To further assess our model, we applied NeuroSCORE to the 27 high-level phenotypes of mouse model data curated by Jackson Labs Mouse Genome Informatics database^18^. Among genes with high-level experimental phenotype data (*N* = 8149), total phenotypes per gene ranged from 1 to 26 with an average of 5.99 phenotypes (SD = 4.55). Using chi squared analysis between high scoring gene orthologs and the presence of any of the 27 phenotypes (Bonferroni corrected for 27 tests, *p* < 0.002), we found high scoring gene orthologs were significantly enriched in seven phenotypes including: mortality and aging, embryonic abnormalities, central and peripheral nervous system abnormalities, growth and congenital anomalies, abnormalities of learning and behavior, abnormalities of cellular proliferation, differentiation, and apoptosis, and abnormal muscle development (all *p* ≤ 2.9e^-5^).

**Figure 3:**
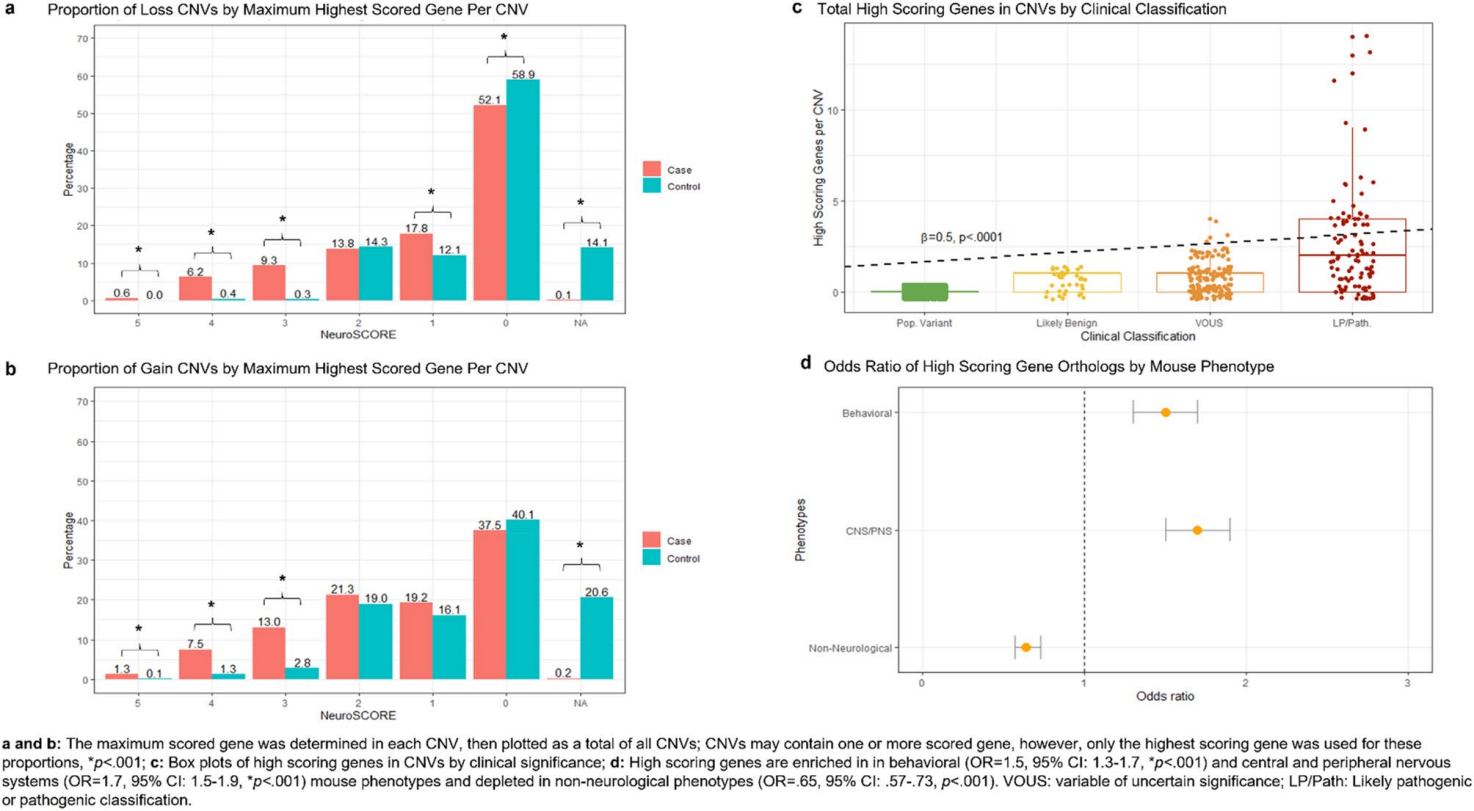
NeuroSCORE applied to human case–control cohorts and mouse phenotype experiments.

Using logistic regression, we found high scoring genes were significantly more likely to be associated with mouse ortholog genes that cause the behavioral phenotypes (OR = 1.5, 95% CI 1.3–1.7, *p* = 5.9e^-10^) and central/peripheral nervous system phenotypes (OR = 1.7, 95% CI 1.5–1.9, *p* = 4.7e^-16^), while they were significantly depleted in genes causing non-neurological phenotypes (OR = 0.64, 95% CI 0.57–0.73, *p* = 3.8e^-12^; see Fig. 3D).

### GO enrichment and pathway analyses

We performed GO Analyses on the set of 1601 high scoring genes to determine if enrichment of key biological processes, cellular components, and molecular functions occurred within this set. Enriched terms in biological processes include positive regulation of protein localization to Cajal body (GO:1904871), axo-dendritic protein transport (GO:0099640), and alternative mRNA splicing, via spliceosome (GO:0000380). Within cellular components, key terms included the nBAF complex (GO:0071565) and the NuRD complex (GO:0016581), which are involved in chromatin remodeling. For the molecular functioning area, terms included binding activity such as protein kinase A catalytic subunit binding (GO:0034236), microtubule plus-end binding (GO:0051010), and pre-mRNA binding (GO:0036002). See Supplemental Table S1 for a list of all of the top five enriched, unique GO annotation terms and high scoring genes for each of the three areas.

From our pathway analyses, we analyzed approximately 500 GO terms with the lowest false discovery rate values (all q-values ≤ 1.3e^-22^) by inspecting the relationships using the AmiGO visualization tool (accessed December 10th, 2020). Within the developmental pathways, the following were enriched: regulation of dendrite development (GO:0050773), regulation of morphogenesis involved in differentiation (GO:0010769), positive regulation of neurogenesis (GO:0050769), and positive regulation of neuron projection development (GO:0010976). Within the metabolic and enzymatic pathways, the following were enriched: regulation of mRNA stability (GO:0043488), regulation of mRNA splicing via the spliceosome (GO:0048024), and catalytic step two of the spliceosome (GO:0071013). Top selected terms and associated genes are presented in Supplemental Table S2. The GO and pathway analyses further support that NeuroSCORE identifies genes in important neurological and developmental processes.

### Enrichment of NeuroSCORE genes in brain-expressed genes

Assuming that genes with elevated brain expression are biologically important for brain growth, development, and/or functioning, we assessed the distribution of genes identified by NeuroSCORE in the Human Protein Atlas. Using chi squared tests, we foudn that NeuroSCORE genes represent a significantly higher proportion of the genes with elevated brain expression for both high scoring and any scoring genes. High scoring genes represent 12.3% (301 of 2442) of genes with elevated expression, compared to 8.5% (1601 of 18,759) for all genes in the genome (χ = 30.5, *p* = 3e^-8^). Comparing genes that received any NeuroSCORE (scores 1–5), we similarly find that genes with elevated brain expression represent 55.3% (1351 of 2442) compared to 44.2% (8298 of 18,759) for all scored genes in the genome (χ = 37.7, *p* = 8e^-10^). This provides additional evidence that NeuroSCORE identified genes relevant to biological function (and likely dysfunction) of the CNS.

### Case–control analyses in neurodevelopmentally affected and typically developing cohorts

After identifying increased ORs for high scoring genes in CNS phenotypes and showing further support for the model through mouse, GO, and brain expression analyses, we next investigated the NeuroSCORE content of human copy number variants (CNV) data. We applied NeuroSCORE to CNV data derived from microarray testing of individuals in both a population control^19^ and a neurodevelopmentally affected cohort^20^. We compared average CNV size, total genes within CNVs, total number of genes identified by NeuroSCORE in the CNVs, and median and average NueroSCORE of the CNV (*p-*values used Bonferroni correction of *p* < 0.005 corresponding to five statistical tests for two classes of CNVs). For easier interpretation of some of the following analyses, NeuroSCOREs were converted from total points to a percentage of total points (0 = 0%, 1 = 20%, 2 = 40%, 3 = 60%, 4 = 80%, and 5 = 100%).

Using two-sided *t-*tests, we found the average size of both loss and gain CNVs was significantly larger in cases than controls (losses: 344 kilobases (kb) vs. 104 kb, *p* = 3e^-8^; gains: 419 kb vs. 244 kb, *p* = 5e^-8^). Within gain CNVs, the average number of genes was similar between cases and controls (4.1 v. 3.6, *p* = 0.1), while there were slightly more genes on average within loss CNVs for cases versus controls (3.2 vs. 2.4, *p* = 0.001). The small range in the average number of genes in case and control CNVs (2.4–4.1 genes) suggests *specific* genes within case CNVs, rather than the total number of genes, could drive the neurodevelopmental phenotypes. Therefore, we investigated the NeuroSCORE profile of these CNVs to assess for differences between cases and controls.

To assess potential differences in gene content, we next analyzed median and average CNV NeuroSCORE and the distribution of scored genes between case and control CNVs. Using two-sided *t*-tests, we found that case CNVs had higher median and average NeuroSCORE (median: losses: 13.4% vs. 6.7%, *p* = 2e^-16^; gains: 14.9% vs. 8.5%, *p* = 2e^-16^; average: losses: 14.2% vs 7.6%, *p* = 2e^-16^; gains: 16.4% vs. 10.5%, *p* = 2e^-16^). To assess the distribution of scored genes within CNVs, we used chi squared analyses using the highest scoring gene in a CNV at each different scoring levels (5, 4, 3, 2, 1, 0, and no score/NA). Using a Bonferroni corrected *p*-value of *p* < 0.004 for 14 tests (7 scoring levels, two classes of CNVs), we found that case CNVs were significantly enriched for the high scoring NeuroSCORE genes in both loss and gain CNVs while controls were enriched for genes that achieved no score (0) in loss CNVs or were not scored/NA in both loss and gain CNVs (Table 3 and Fig. 3A, B). In case CNVs, 16.2% of losses and 21.8% of gains contained at least one high scoring gene compared to 0.6% and 4.2% of controls, respectively. Logistic regression showed a significantly increased OR for case CNVs containing one or more high scoring genes compared to controls (OR = 9.3, 95% CI 7.4–11.8, *p* = 2e^-16^). Taken together, these results support the ability of NeuroSCORE to identify differences in CNVs associated with neurodevelopmental features compared to population controls.

**Table 3:** Distribution of the highest scored gene within case and control CNVs.

NeuroSCORE	Genes in loss CNVs			Genes in gain CNVs		
	Cases *N* (%)	Controls *N* (%)	*p*	Cases *N* (%)	Controls *N* (%)	*p*
5	5 (0.6)	0 (0)	9e^-4^	18 (1.3)	1 (0.1)	1e^-6^
4	52 (6.2)	9 (0.4)	2e^-16^	102 (7.5)	25 (1.3)	2e^-16^
3	78 (9.3)	7 (0.3)	2e^-16^	176 (13.0)	52 (2.8)	2e^-16^
2	115 (13.8)	365 (14.3)	.73	288 (21.2)	354 (19.0)	.13
1	149 (17.8)	307 (12.1)	2e^-5^	261 (19.2)	300 (16.1)	.68
0	435 (52.1)	1501 (58.9)	6e^-4^	509 (37.5)	746 (40.1)	.15
NA	1 (0.1)	358 (14.1)	2e^-16^	3 (0.2)	384 (20.6)	2e^-16^

NA are genes that could not be scored (e.g., pseudogenes); total case CNVs N_LOSSES_ = 835, N_GAINS_ = 1357, total control CNVs N_LOSSES_ = 2547, N_GAINS_ = 1862; For statistical testing, Fisher’s Exact test was used for analyses when genes in CNVs were ≤ 5, while Chi squared tests were used for analyses when genes in CNVs were > 5; significance set at *p* ≤ 4e^-3^ after Bonferroni correction for 14 tests.

We next performed sub-analyses of case–control CNV by gender (N_MALES_ = 1724, N_FEMALES_ = 468), inheritance (N_PATERNAL_ = 35, N_MATERNAL_ = 43, N_*de novo*_ = 33), and clinical classification. Classifications included common variants (“population”) and those classified as likely benign, variants of uncertain clinical significance (VOUS), and clinically significant (N_POPULATION_ = 2195, N_LIKELY BENIGN_ = 39, N_VOUS_ = 148, N_CLIN. SIG_ = 96; Fig. 3C).

Previous work has shown that the gender bias in neurodevelopmental conditions is partly due to the burden of CNVs or sequence variants^21^. Given this, we questioned if affected females had CNVs with higher NeuroSCOREs than affected males. Analyzing case CNVs by gender showed no differences for average NeuroSCORE (15.1% vs. 15.7%), median NeuroSCORE (13.7% vs. 14.6%), or rates of high scoring genes within CNVs (0.4 vs. 0.3). Similarly, we did not find differences in CNVs by inheritance (inherited vs. de novo) for average NeuroSCORE (20.6% vs. 26.1%), median NeuroSCORE (17.3% vs. 25.2%), or rates of high scoring genes (1.8 vs. 0.7). However, using linear regression and controlling for CNV size we found that CNVs with increasing pathogenicity showed an increase in the total number of high scoring genes (*β* = 0.5; *p* = 2e^-16^) and lower scoring genes (*β* = 0.02; *p* = 0.02), while non-scored genes decreased slightly (*β* = −0.05; *p* = 2.5e^-8^, Fig. 3C). As expected, linear regression also showed median CNV NeuroSCORE increased with increasing classification as well (*β* = 6.7%; *p* < 2 e^-16^). These data show high scoring genes appear to be an important component in CNVs identified in this clinically affected cohort.

### Landscape of high scoring genes in pathogenic CNVs

**Figure 4:**
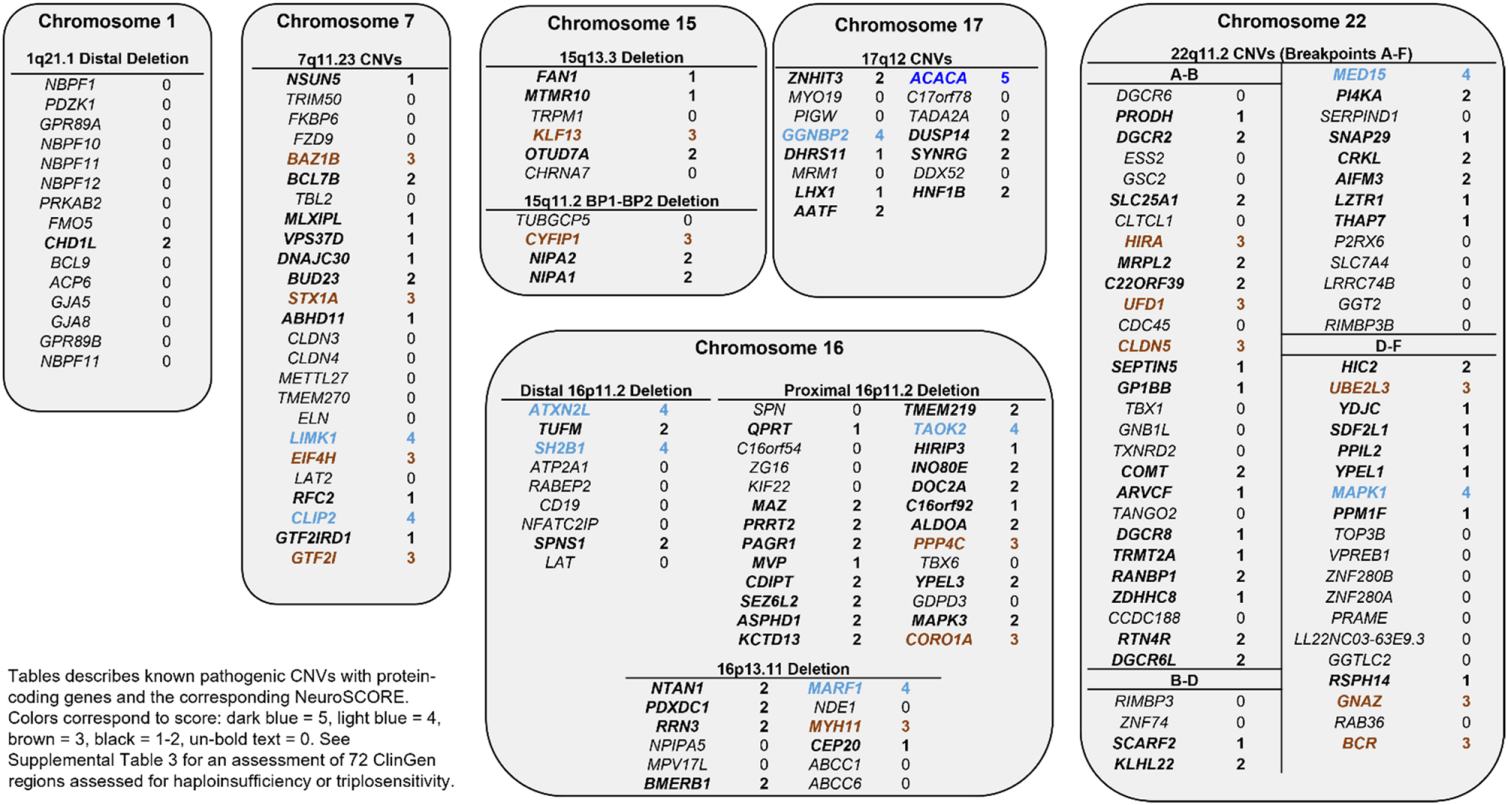
Common neurodevelopmental microdeletion/Duplication syndromes with gene level NeuroSCORE.

Given pathogenic CNVs underlie a significant proportion of many neurological disorders, we applied NeuroSCORE to a set of common pathogenic CNVs as described in > 10,000 individuals with neurological or neurodevelopmental phenotypes^22^. All CNVs had at least one scored gene and all but the 1q21.1 distal deletion CNV had at least one high scoring gene (Fig. 4).

We next applied NeuroSCORE to the 72 CNV regions with a completed haploinsufficiency and triplosensitivity review in ClinGen, the Clinical Genome Resource which provides expert curation of the clinical relevance of genes and genetic variants (accessed January 20, 2021, Supplemental Table S3). There are currently 38 regions designated by ClinGen as having sufficient evidence for haploinsufficiency and 21 regions designated with sufficient evidence for triplosensitivity. Although some regions may vary in size, we used the coordinates provided by ClinGen (hg19/GRCh37). The majority of these regions contain at least one gene with a high NeuroSCORE (30/38 and 14/21, respectively). Next, we explored the NeuroSCORE profile of several recurrent CNV regions to help identify genes that are likely to contribute to CNS-related phenotypic features.

The 7q11.23 recurrent region is approximately 1.5 megabases with deletions associated with Williams-Beuren syndrome (WBS) and gains associated with 7q11.23 duplication syndrome. The typical WBS deletion/duplication region contains 25 total genes, of which 15 are scored and six are high scoring genes. The *GTF2I* and *GTF2IRD1* genes have been implicated as key genes driving the neurobehavioral phenotype^23,24^, though *GTF2I* and *GTF2IRD1* knockout mice suggests that neither gene fully recapitulates the neurobehavioral aspects of WBS^23^. Aside from *GTF2I,* NeuroSCORE identified five high scoring genes (Fig. 4), all with evidence for CNS involvement: *STX1A* has been associated with ASD^25^ and syndromic ID^26^ in humans, *LIMK1* sequence variants are associated with ASD^27^ and visuospatial impairment^28^, while *LIMK1* deficient mice have fewer cortical pyramidal neurons^29^; *BAZ1B* knockout mice show abnormal neurogenesis^30^ while clinical studies show variants associated with ASD^27^, Klippel-Feil syndrome^31^, and congenital heart defects^27^; *EIF4H*-deficient mice have a smaller body size, behavioral impairments, and reduced brain volume^32^; finally, a single *CLIP2* variant has been associated with ASD^33^.

The 22q11.2 region is also associated with recurrent deletion and duplication syndromes. Within the typical 22q11.2 region there are 64 genes, of which 38 are scored and eight are high scoring genes. However, the 22q11.2 deletion/duplication syndrome region presents a challenge when interpreting CNVs that are smaller than the common breakpoint in the A-F deletion (breakpoints refer to areas of repetitive DNA segments that cause recurrent CNVs and often are given letter designations). Our analysis found the 22q11.2 A–B, B–D, and D–F breakpoint CNVs each contain multiple scored genes and at least one high scoring gene (Fig. 4), suggesting that CNVs of any of these smaller regions of 22q11.2 may be pathogenic for CNS-related clinical features. Two previous studies in cohorts of individuals with 22q11.2 deletion syndrome are also consistent with our data. The first study analyzed neuroimaging and transcriptomic data and identified the *MAPK1* gene (a gene in the D-F region with NeuroSCORE of 4) as a potential driver gene of brain morphology changes^34^. The second study analyzed 22q11.2 deletion size and IQ score, finding that IQ score was partially explained by deletion size (A–B vs. B–D)^35^. Taken together, NeuroSCORE identified several candidate genes in the 7q11.23 and 22q11.2 regions, some of which are supported by other studies as well as additional genes that may provide new insight into the role of these regions in neurological phenotypes.

Emerging CNV syndromes also pose challenges for clinical interpretation. Applying NeuroSCORE to rare CNVs can help determine if they score in the same range as known pathogenic CNVs while also identifying candidate genes. We queried the ClinGen Dosage Sensitivity Map and identified the 2p16.1-p15 CNV (chr2:54,700,000–63,900,000; hg19) as one with limited data on whether it caused a duplication syndrome. To date, seven individuals have been reported with a duplication and neurological clinical features^36–38^. Experimental studies in zebrafish of the previous candidate genes have implicated the *BCL11A, USP34, REL,* and *XPO1* genes^39^ as possibly associated with a phenotype. Using NeuroSCORE, we first find this CNV has a median score of 30%, well above the average median score of 16% in gain CNVs in our affected case cohort. Furthermore, within this region are four high scoring genes (*BCL11A, USP34, XPO1,* and *CCT4*), of which the *CCT4* gene was not identified by previous studies. Experimental work in *Drosophila* shows *CCT4* knockdown results in severely reduced dendritic growth^40^; furthermore, two de novo, missense variants have been reported in two individuals with ASD^27^. These data support a role for 2p15-p16.1 gains in CNS phenotypes and indicate the *CCT4* gene as a new candidate gene.

## Discussion

We hypothesized that CNS-related disease genes are likely to be identified by multiple metrics that assess different genic properties. After combining multiple genome-wide databases that assess different properties, we found that five metrics were independently and positively associated with genes already known to cause CNS-related conditions as identified in the OMIM database. These five metrics have similar odds ratios in logistic regression modeling (range: 1.4–2.2) and suggest that they have similar strengths of association with known neurogenetic disease genes and that no single variable has an outsized influence on the model. A total of 8298 genes were identified by at least one of five gene metrics and 1601 genes were identified by three or more. These high scoring genes more often have elevated gene expression in the human brain, are enriched in developmental and neurological pathways, show neurologically related phenotypes in murine experiments, and are affected by CNVs more often in a neurodevelopmentally-affected cohort (Table 3 and Fig. 3). Conversely, genes that scored 0 in our model were enriched for genes that are associated with non-CNS-related phenotypes. Of these 1601 high scoring genes NeuroSCORE identified, 1058 (66%) do not yet have any phenotype association in OMIM (accessed July 31st, 2021) and 210 genes (13%) have no associated variants in the Human Genome Mutation Database (HGMD; accessed November 11th, 2021).

When we transform the logistic regression results of high scoring genes to the logistic likelihood curve, we can determine the likelihood that any gene within the set will cause a condition with CNS-related features. Transforming these results shows that genes within this set have an 84–92% chance of being associated a condition that results in CNS-related features, suggesting the substantial majority of the 1601 genes are likely to eventually be associated with neurogenic disorders. These 1601 gene likely represent a proportion of undiscovered neurodevelopmental genes proposed by recent analysis^9^. However, NeuroSCORE also captures genes related to neuropsychiatric or neurodegenerative disease. Analyses of genes associated with phenotypes such as ataxia, Parkinson’s, and amyotrophic lateral sclerosis/frontotemporal dementia (ALS/FTD) showed the majority of genes are scored (ataxia: *N* = 4 of 5; Parkinson’s: *N* = 19 of 21, and ALS/FTD: *N* = 26 of 32). Further, many fall into the high scoring range (ataxia: *N* = 2 of 5; Parkinson’s: *N* = 7 of 21, and ALS/FTD: *N* = 8 of 32). Building on the NeuroSCORE framework to target more specific phenotypes may further broaden its applicability in the future.

The findings from our pathway and GO analyses found significant enrichment in multiple neurological processes, many with known disease or phenotype associations (Supplemental Tables S1 and S2): the BAF (or SWI/SNF) complex^41^, the NuRD complex^42^, neuronal organization with microtubule tracking^43^, tau protein activity^44^, histone binding processes^45^, the Cajal body^46,47^, proteasome activity^48^, mRNA processing via both the spliceosome components^49^ and mRNA trafficking and binding^50^. Of these processes, splicing may be one of the most important. Tissue-specific splicing in the brain has shown high rates of alternative transcript splicing, suggesting that splicing proteins and proper splicing are imperative to neuronal development, structure, and function and appear to be evolutionarily conserved^51^. The Cajal body presents an interesting confluence of multiple previously discussed CNS-disease related processes as these nuclear bodies appear in fetal and neural cells to help mediate splicing, create parts of the spliceosome and ribonuclear proteins, activate transcription, and aid in chromatin and genome organization^46^. Considering that ASD and other neurodevelopmental disorders appear to begin in the fetal period^52^, the enrichment of Cajal body-associated genes in our analyses raises an interesting target for additional study of genes related to possible “Cajalopathies”.

Our model identified 123 high scoring genes that are associated with conditions in OMIM that have no known CNS-related clinical features (OMIM accessed July 31st, 2021). While some of these genes are likely false positives and will not be found to cause CNS-related features, some have emerging evidence for causing CNS-related phenotypes. One example is *MORC2*, currently associated with only a form of Charcot-Marie-Tooth disease (OMIM #616661) but recently reported to cause a neurodevelopmental disorder^53^. Using NeuroSCORE could help identify CNS disease genes that have been overlooked due to prior disease associations (see Supplementary Table S4 for a list of genes).

Finally, our model may be helpful to identify genes that influence or increase the risk for spectrum conditions, such as ASD. Multiple damaging variants in multiple scored genes could explain the risk or presence of a condition like ASD in individuals without a known variant in high-risk genes or common pathogenic CNVs. Damaging variants in scored genes may also help explain variable expressivity and reduced penetrance observed in many CNS-related genetic conditions (e.g., “two-hit” models).

Like Amirah Brigham’s and John Snow’s use of mapping in the nineteenth century cholera epidemic, we have correlated existing data and created a new map to aid in discovery of conditions that broadly effect humanity. Our NeuroSCORE map of the human genome identifies and prioritizes potential disease genes of the CNS, which we validated using case–control and mouse model organism data. NeuroSCORE can be used for bioinformatic analysis pipelines, identification of candidate disease genes in individuals with neurological phenotypes, guidance of basic and clinical research, the development of genetic tests, and furthering research on treatments for these conditions as current or future medications may target specific proteins or pathways. Future directions of model development can include identifying interaction terms to improve model precision as well as adding new metrics such as transcriptome or methylation profiles from brain expressed genes. While there are genes that cause CNS-related conditions not identified by NeuroSCORE (e.g., *GABRG2*), our model represents a potentially significant step forward in research to improve ultimately diagnostics for individuals with genetic causes of neurological conditions.

### Limitations

One limitation to this study and model is that it analyzes only protein-coding genes and excludes disease mechanisms such as mitochondrial, epigenetic, and disruption of enhancer and untranslated regions. Recent work in a small ASD cohort indicates that certain neurological conditions, such as ASD risk, may be influenced by variants in non-coding regions^54^. Similarly, genes causing autosomal recessive conditions are not well represented due to the use of gnomAD loss-of-function data. However, a recent analysis in the Deciphering Developmental Disorders cohort found approximately 3.6% of individuals from non-consanguineous families had a condition consistent with a recessive inheritance pattern^55^. Phenotypes caused by a significant proportion of environmental factors or oligogenic risks are similarly not well identified using NeuroSCORE. Another limitation is that our outcome variable (CNS clinical features) is drawn from OMIM, which is a rigorously maintained database but also an imperfect store of genotype–phenotype information due to possibly inaccurate or outdated information and ascertainment bias. Finally, many conditions are not yet fully phenotyped, with rare phenotypes or age-related phenotypes not well represented.

## Methods

### Building the NeuroSCORE model

To build a comprehensive and clinically useful model, we chose genome-wide datasets with gene-specific data and combined them. As this analysis focused on protein-coding genes, we excluded non-coding genes, RNA-coding genes, genes in the mitochondrial DNA, and pseudogenes. We sought lines of evidence previously associated with neurodevelopmental or neurological phenotypes including loss of function constraint^11,13^, constrained coding regions^12^, de novo variation^27,56–60^, brain expression levels^61^, copy number variation^58,62^, and genes with exons that are both highly expressed in brain tissues and under purifying selection^63^. If a gene was identified by one of the following metrics, it received a score of one point (a categorical variable, yes vs. no). We began with seven preliminary gene metrics (possible independent variables) from which to build our model. Of note, two of these metrics have two levels—one more restrictive than the other—yielding nine total possible variables from which to begin exploratory analyses. Of these nine, only five were retained in our final model, as described in the Results (Fig. 1) and designated by an asterisk (*) below:

1. gnomAD LOF*: The gene’s upper bound score of the gnomAD observed/expected (o/e) loss-of-function metric was < 0.35, the preferred cutoff stated on the gnomAD site^13^, with rounding from the thousandths place (i.e., genes below 0.345). Using gnomAD v2.1 (accessed May 2019), there were 2896 genes identified by the gnomAD LOF gene metric.
2. Critical Constraint*: The gene contained at least one area of regional constraint (critically constrained regions; CCRs) at or above the 95th or 99th percentile as described by Havrilla et al*.*^12^. The CCR 95 and CCR 99 gene metrics identified 7049 and 1444 genes, respectively, and were chosen as cutoffs as suggested in the original paper. Only genes identified at the 99th percentile were included in the final model.
3. GTEx*: The median brain expression across the 13 brain tissues assessed by the Gene-Tissue Expression database v8 was ≥ 10 transcripts per million (“GTEx genes”)^64^. This cutoff was chosen based on the recommendation of the European Bioinformatics Institute (https://www.ebi.ac.uk/gxa/FAQ.html). The GTEx gene metric identified 6069 genes.
4. De novo*: The gene was enriched for de novo variants as reported in the de novo Database using the non-Simon Simplex Cohort data (assessed January 17th, 2020)^65^. Regardless of potential variant pathogenicity, variants within protein-coding genes or the 3’ or 5’ untranslated regions from 13,168 trio or quartet exome/genome probands were collated from 31 unique studies for the following phenotypes: epilepsy, ASD, developmental delays, cerebral palsy, bipolar disorders 1 and 2, schizophrenia, early-onset Alzheimer and Parkinson disease, intellectual disability, neural tube defects, sporadic infantile spasm syndrome, and Tourette syndrome (see Supplementary Table S5 for a description of all studies used). We adopted a conservative approach to define a gene enriched with de novo variants if the genes contained ≥ 10 reported de novo variants. The de novo gene metric identified 487 genes.
5. Index*: The gene was identified by a previous exon indexing tool (“Index genes”) with exons expressed at or above the 90th percentile in 388 post-mortem brain samples and below the 10th percentile in mutational burden for rare (< 5%) missense or loss-of-function variants in the 1000 genomes database^63,66^. Although specific exons within a gene are identified with this tool, we scored the entire gene if ≥ 1 exon in the gene was identified. (Note: The cutoffs differ from those originally reported in the Uddin et al.^63^ paper as they are more stringent and are used by Lineagen, Inc. in interpretation of clinical testing.) The Index gene metric identified 4646 genes.
6. gnomAD MIS: The gene’s upper bound score of the gnomAD o/e missense variant metric was < 0.35 (the preferred cutoff stated on the gnomAD site)^13^. The gnomAD MIS gene metric identified 112 genes. Ultimately, this gene set was not used in our model (see “Results”).
7. Coe et al*.*: The gene was enriched in the Coe et al.^62^ case–control study of individuals with childhood developmental conditions and CNVs. This study was used as it has gene-level statistics on enrichment in a neurodevelopmentally affected cohort. We used two cutoff points for the Coe gene metrics based on two significance values: *p* ≤ 0.01 (Coe 1 = 3116 genes) or *p* ≤ 0.02 (Coe 2 = 3732 genes). These *p*-value cutoffs were used historically in our clinical testing to determine genes or regions of potential clinical relevance. Ultimately, Coe et al*.* data were not used in our final model (see “Results”).

Our outcome (independent) variable was defined as whether or not a gene was associated with a phenotype containing one or more CNS-related clinical features in OMIM; this was also categorical variable (1 vs. 0, yes vs. no) and was assessed regardless of mode of inheritance. We used a previous definition of the CNS as including only the brain and spinal cord^67^. While an exhaustive list of CNS phenotypes meeting inclusion criteria are not possible here, examples include developmental or psychomotor delays, specific delays (e.g., speech), developmental regression, intellectual disability (or “mental retardation”), ASD or autistic traits, para- or diplegias, seizures or epilepsy, EEG abnormalities, structural anomalies of the brain or spinal cord, hydrocephalus, altered pain tolerance, sleep abnormalities, movement or coordination disorders, ataxia, tone abnormaltieis, behavioral abnormalities (e.g., aggression), psychiatric disorders, hallucinations, personality changes, emotional lability, hyper- or areflexia, Parkinson’s disease, Alzheimer’s disease, frontotemporal dementia, amyotrophic lateral sclerosis, and others. We excluded phenotypes affecting the eye, retina, cochlea, and peripheral nervous system, or conditions that caused CNS involvement due to an external event (e.g., thromboembolism). Metabolic conditions and mitochondrial conditions caused by nuclear genes were included as having CNS phenotypes as the cellular dysfunction leading to symptoms originates within the cells of the CNS. Although the retina is derived from the CNS, phenotypes involving only the retina were excluded as these conditions are treated clinically as ophthalmological conditions. Two authors (KD and MR) reviewed the top 13,021 genes in OMIM (ranked by median brain expression) which included 1822 genes with a phenotype including CNS-related clinical features and 1513 genes with phenotypes that did not have CNS-related clinical features.

### Merging databases and data fidelity

Due to genes having multiple historic names, we matched data between databases by both gene name and Ensemble ID. One author (KD) visually inspected all genes identified by the primary data sources and cross-referenced discrepant or missing data with external databases (e.g., HUGO) to ensure data was present. If a gene name was discrepant, the name was updated to the current HGNC-approved name. In total, our model assesses 19,601 genes, while data for 842 genes was not available from at least one gene metric; these genes were scored as “NA”.

### Statistical analyses of the NeuroSCORE model

We first assessed each of the seven gene metrics and their association with genes currently known to cause or contribute to CNS-related neurological phenotypes. We initially performed Pearson’s chi squared on each gene metric then included the variables significant at *p* < 0.05 in a multiple logistic regression model. Nine total variables were assessed (seven metrics with the Coe and CCR gene metrics having two levels). Using SAS v9.4, we constructed a multiple logistic regression model with backward elimination to remove variables with high multicollinearity or those that were not associated with CNS-related clinical features at *p* < 0.05. Wald testing was used to determine if each of the variables in the final model were significantly different from zero.

Using multiple logistic regression, we examined main effects and determined the odds ratios (ORs) for the final five metrics to be associated with genes known to cause CNS-related clinical features. We measured ORs for each variable in the final NeuroSCORE model as well as genes identified by multiple metrics (NeuroSCOREs 2–5). Both analyses used a comparison group of 4723 genes that were not identified by any metric (NeuroSCORE of 0). This yielded 1133 genes with a score of 0 that were linked to any known phenotype in OMIM (through June 2020). We performed a power analysis for these genes with a NeuroSCORE of 1 to determine the minimal detectable OR given our sample size. Setting *β* = 0.95 and *α* = 0.05 for this group of genes, the minimum OR we could detect was 1.4. All odds ratios were calculated using R using v.1.2.1335 or SAS v9.4; power analyses were performed in R with the EpiR package v2.0.17.

### Evaluation of NeuroSCORE model in real-world case and control cohorts

We used two published cohorts to evaluate NeuroSCORE. As exome analyses are often performed with priority to genes already known to be involved in genetic conditions, we used copy number variant (CNVs) from microarray data from individuals affected with neurological conditions and population control cohorts. This is because CNVs often contain multiple genes of known and unknown function and significance. We matched all genes in all included CNVs to their corresponding NeuroSCORE by gene name and visually inspected and corrected all discrepancies. CNVs with only non-scored genes (e.g., pseudogenes) were omitted from analysis.

The population control cohort was drawn from the Ontario Population Genomics Platform reporting on CNVs from 1000 adults, providing 6965 total CNVs^19^. After removing CNVs that did not affect at least one exon of one gene, the control cohort contained 1862 gain CNVs and 2547 loss CNVs. For the case comparison group, we began with a previously published cohort of 2691 individuals with neurodevelopmental conditions including ASD, schizophrenia, attention deficit hyperactivity disorder (ADHD), and obsessive–compulsive disorder^20^. Almost half of this total cohort (46%, 1230/2691) was assessed for intellectual disability (ID), of which 14.9% (183/1230) received the diagnosis and thus had ID combined with ASD, schizophrenia, or ADHD. We used CNVs consistently identified by multiple CNV calling algorithms (“stringent” CNVs) and were identified either as “rare” (< 0.1% in a control population) or being deemed of possible clinical relevance (see Table 2 in Zarrei et al*.*^20^). After removing CNVs from 17 individuals with aneuploidies, the final case data contained 1357 gain CNVs and 835 loss CNVs. We included inheritance and clinical classification data when available. Both cohorts were assessed using the Cytoscan HD microarray platform with the same CNV calling algorithms and similar sizing and probe cutoffs.

For each CNV, we paired every gene with its respective NeuroSCORE, then generated a median and average NeuroSCORE for the CNV. To simplify some of these analyses, we converted NeuroSCOREs to percentages of the total possible points (1 = 20%, 2 = 40%, 3 = 60%, 4 = 80%, 5 = 100%). We used two-sided *t*-tests to compare differences in CNVs between cases and controls for CNV size, total gene content, and the average and median NeuroSCORE. We then used Pearson’s chi squared or Fisher’s exact test (if *N* ≤ 5) to compare the distributions of the maximum scored gene within case and control CNVs. We performed sub-analyses using two-sided *t*-tests to explore differences in NeuroSCORE and scored genes by inheritance (inherited vs. de novo), gender (male vs. female proband), and clinical significance (common population variants, likely benign variants, variant of uncertain significance (VOUS), and likely clinically significant/clinically significant).

Finally, we performed linear regression analysis on classification and gene content by assigning increasing values to increasing pathogenicity and using the total count of genes in the CNV (see “Results”, Fig. 3 and case–control analyses). Zarrei et al*.*^20^ classified CNV pathogenicity, though we added a classification for common, “population variants” (CNVs observed at > 1% in the cohorts). Classification was coded as 1 = population variant, 2 = likely benign, 3 = VOUS, 4 = pathogenic/likely pathogenic.

### NeuroSCORE in brain-expressed genes from the human protein atlas

Using the Human Protein Atlas, we analyzed genes defined as having “elevated” brain expression (*N* = 2587 genes), corresponding to gene expression within a brain region or cell type > 4 × higher than genes expressed in another tissue or cell type^68^. Using this set of genes, we compared the distribution of high scoring and any scoring genes to genome-wide NeuroSCORE data to determine if scored genes were enriched in this data (retrieved November 3, 2021 from https://www.proteinatlas.org/humanproteome/brain). After removing 145 genes due to not being a protein coding gene or a lack of scoring data, our data set included 2442 genes for analysis. We used chi squared tests to determine if the distribution of high scoring genes or any scored gene in the “elevated” brain expression data set was different than the distribution of these genes in the genome.

### GO and pathway analyses

Gene ontology (GO) enrichment analysis was performed for the set of high scoring genes (identified by 3 or more gene metrics in our final model; *N* = 1601)^69–71^. We performed analyses for biological processes, molecular function, and cellular component using Bonferroni correction for multiple testing. We chose this correction as it is the most conservative correction. If multiple related terms were within the top enriched GO terms, we included the more specific term and omitted the broader term. This analysis used databased from the Gene Ontology Consortium (http://geneontology.org/) using the March 23rd, 2020 release.

To map the relevant pathways in which the high scoring genes were primarily involved, gene enrichment analysis was performed using the gene overlap package of R, followed by Cytoscape analysis to trace the pathways involved and their connectivity. The false decision rate and *p*-value cut off was 0.01 and 0.001, respectively. Kyoto Encyclopedia of Genes and Genomes (KEGG) and Gene Ontology (GO) database were used for both gene enrichment and Cytoscape analysis. Then, the network was built using the enrichment map and the auto annotate Cytoscape application. The node color represents the *p*-value (the darker the shade, the lower the *p*-value) and size of the node represents increasing odds ratio.

### Evaluation of NeuroSCORE model in mouse model data

We used the high-level phenotypic data provided by the Jackson Laboratory’s Mouse Genome Informatics database to further assess our model (accessed July 6th, 2020; http://www.informatics.jax.org)^18^. We downloaded all annotated genes and matched the mouse and human gene using the unique MGI number via HGNC database. We excluded genes where the human homolog of the mouse gene included two or more unique human genes (e.g., the mouse gene *Rln1* is a homolog of both human *RLN1* and *RLN2*). We also removed multiple mouse genes that matched the same human ortholog (e.g., mouse genes *SCD1*, *SCD2*, *SCD3*, and *SCD4* are homologues of human *SCD*). Lastly, we removed genes where no mouse phenotype information was available, as a lack of high-level phenotype information does not mean variants of a gene cannot cause a phenotype. In total, we removed: 70 genes with multiple mouse homologues matching to a single human gene, 45 human genes that did not have a mouse ortholog, and 4139 genes without phenotype data. The total number of genes included for analysis with phenotype data was 8149, which included 3863 genes associated with any neurological phenotype and 4286 genes associated with any non-neurological phenotype. Of note, this data was not used to develop or refine the NeuroSCORE model.

## Supplementary Information

Supplementary Information 1.

Supplementary Information 2.

Supplementary Information 3.

Supplementary Information 4.

Supplementary Information 5.

## Data Availability

The data that support the findings of this study are available from Bionano Genomics, Inc., but restrictions apply to the availability of these data, which were used under license for the current study, and so are not publicly available. Data, however, may be available from the authors upon reasonable request and with permission of Bionano Genomics, Inc.
