# Supplementary material for: NeuroSCORE is a genome-wide omics-based model that identifies candidate disease genes of the central nervous system
Source: Sci Rep. 2022 Mar 31;12:5427. doi: 10.1038/s41598-022-08938-y (PMC8971396; doi:10.1038/s41598-022-08938-y)
Supplement: Supplementary file 1 — Supplementary Information 1. [file 41598_2022_8938_MOESM1_ESM.pdf]

**Supplemental Table S1: Top Five Enriched Gene Ontology Terms & Candidate OMIM Genes Among High Scoring Genes**

| GO Annotation Term | Genes (N) | Genes Expected (N) | Enrichment | Genes without OMIM CNS-Related Phenotypes |
|---|---|---|---|---|
| **Biological Processes** | | | | |
| Positive regulation of protein localization to Cajal body (GO:1904871) | 9 | 0.7 | 13.0* | ***CCT2, CCT3, CCT4, CCT7, CCT8, CCT6A, DKC1, TCP1*** |
| Positive regulation of establishment of protein localization to telomere (GO:1904851) | 9 | 0.8 | 11.7* | ***CCT2, CCT3, CCT4, CCT7, CCT8, CCT6A, DKC1, TCP1*** |
| Axo-dendritic protein transport (GO:0099640) | 10 | 1.0 | 10.0* | *DLG2, KIF5B, MAP1A, TERF2,* ***SFPQ*** |
| Alternative mRNA splicing, via spliceosome (GO:0000380) | 13 | 1.3 | 9.9*** | ***CELF4***, ***DDX5***, *DDX17, DHX9, HNRNPM, RBM17,* ***SFPQ,*** *SFSWAP,* ***SLU7,*** *SRSF1* |
| Positive regulation of telomerase RNA localization to Cajal body (GO:1904874) | 11 | 1.2 | 9.5** | ***CCT2, CCT3, CCT4***, ***CCT7, CCT8, CCT6A, DKC1,*** *RUVBL1, RUVBL2,* ***TCP1*** |
| **Cellular Component** | | | | |
| nBAF complex (GO:0071565) | 12 | 1.2 | 10.4*** | *DPF1, SMARCC1* |
| Proteasome regulatory particle, base subcomplex (GO:0008540) | 9 | 0.9 | 9.8** | ***PSMC1, PSMC2, PSMC3, PSMC4, PSMC5, PSMC6, PSMD1, PSMD2, PSMD4*** |
| Chaperonin-containing T-complex (GO:0005832) | 8 | 0.9 | 9.5* | ***CCT2, CCT3, CCT4, CCT7, CCT8, CCT6A, TCP1*** |
| NuRD complex (GO:0016581) | 11 | 1.2 | 8.9** | *MTA3, HDAC2, RBBP7, MBD3, ZBTB7A, MTA1, CHD5, RBBP4* |
| Proteasome regulatory particle (GO:0005838) | 15 | 1.7 | 8.9*** | ***PSMC4, PSMC6***, *PSMD6, PSMD14,* ***PSMD2***, *PSMD11,* ***PSMC3***, ***PSMC1***, *PSMD13,* ***PSMC2***, *ADRM1,* ***PSMD1***, *PSMD3,* ***PSMD4, PSMC5*** |
| **Molecular Function** | | | | |
| Protein kinase A catalytic subunit binding (GO:0034236) | 10 | 1.0 | 10.0** | *CSK, EZR,* ***GSK3B, GSK3A***, *PRKAR1B, PRKAR2B, PJA2* |
| Tau-protein kinase activity (GO:0050321) | 14 | 1.7 | 8.3** | *TTBK1,* ***GSK3B***, *TAOK2,* ***GSK3A***, *BRSK1, MARK4, FYN, BRSK2, ROCK2, TAOK1, MARK2* |
| Microtubule plus-end binding (GO:0051010) | 10 | 1.2 | 8.1* | *CLASP1, CLASP2, CLIP1, CLIP2, MAPRE1, MAPRE2, NUMA1* |
| Pre-mRNA binding (GO:0036002) | 21 | 2.9 | 7.4*** | *CELF1, CELF2,* ***CELF4,*** *CELF5,* ***DDX5***, *HNRNPL, PRPF8, RBM22, RBM4, SF1, SRSF2,* ***SLU7***, *TRA2B, U2AF2* |
| Lysine-acetylated histone binding (GO:0070577) | 11 | 1.5 | 7.2* | *CARM1, BRD2, BRD3, BRD4, BRD7, ZMYND8* |

$*p \leq 0.05$, $**p \leq 0.01$, $***p \leq 0.001$; *p*-values for Chi-squared testing uses Bonferroni correction for multiple testing for Biological processes (9,050 unique tests), Cellular Component (1,472 unique tests), Molecular Function (2,811 unique tests); **bold** genes appear two or more times in this table
