# Supplementary material for: NeuroSCORE is a genome-wide omics-based model that identifies candidate disease genes of the central nervous system
Source: Sci Rep. 2022 Mar 31;12:5427. doi: 10.1038/s41598-022-08938-y (PMC8971396; doi:10.1038/s41598-022-08938-y)
Supplement: Supplementary file 2 — Supplementary Information 2. [file 41598_2022_8938_MOESM2_ESM.pdf]

Supplemental Table S2: Select Pathways and Candidate Genes from AmiGO Visualization

GO Term	FDR	Genes without OMIM CNS-Related Phenotypes
Regulation of dendrite development (GO:0050773)	4.1E^{-23}	***ACTR2,*** *ADGRB3*, ***ANAPC2, BAIAP2***, ***CAMK1D,*** *CAMSAP2*, ***CAPRIN1,*** *CARM1*, ***CRK, CYFIP1, DAB2IP, DBN1,*** *GSK3A*, *GSK3B*, ***PARP6, PREX1, STK11,*** *YWHAH*
Regulation of morphogenesis involved in differentiation (GO:0010769)	8.0E^{-34}	*ACTN4*, ***ACTR2***, ***ANAPC2,*** *ARHGEF7*, *ARPC2*, ***BAIAP2***, ***CAPRIN1***, *CORO1C*, ***CRK***, ***DBN1,*** *DMTN*, *P4HB*, ***PARP6, PREX1***, *PTK2*, ***PTPRD,*** *RCC2*, *TESK1*
Positive regulation of neurogenesis (GO:0050769)	1.9E^{-27}	***ACTR2***, *AMIGO1*, ***ANAPC2, BAIAP2***, *BMPR2*, ***CAPRIN1, CYFIP1, DBN1***, *ETV5*, *FN1*, *HDAC2*, *HIF1A*, *LIMK1*, ***LRP1***, *NAP1L1*, ***NDEL1, NPTN***, *NUMBL*, ***PARP6***, *PLXNB1*, ***PLXNB2***, *PPP1CC*, *PRMT5*, ***PRPF19, PTPRD,*** *PTPRZ1*, *RAB11A*, *RELA*, *RHEB*, ***SNW1***, *SPEN*, *SRF*, ***STK11***, *TTBK1*, ***TWF2***, *XRCC5*
Positive regulation of neuron projection development (GO:0010976)	1.8E^{-22}	***ACTR2,*** *AP2A1*, *APBB1*, *ARHGAP35*, ***BAIAP2, CAMK1D***, ***CAPRIN1, CYFIP1***, ***DAB2IP, DBN1,*** *DPYSL3*, *FYN*, ***LRP1,*** *MARK2*, ***NDEL1,*** *NDRG4*, ***NPTN***, ***PLXNB2***, *PPP2R5B*, *PTK2B*, *RAP1A*, *RAPGEF1*, ***SF3A2***, *STMN2*, ***TWF2***
Regulation of mRNA stability (GO:0043488)	5.8E^{-30}	*ANP32A*, ***CELF1***, *DHX9*, *EIF4ENIF1*, *ELAVL1*, *FASTK*, ***FXR1***, ***FXR2,*** *HNRNPA0*, ***HNRNPC***, *HNRNPD*, ***HNRNPM***, ***HNRNPR***, ***HSPA8***, *KHSRP*, *LARP1*, *MAPKAPK2*, *NPM1*, ***PABPC1***, *PABPC4*, *PAIP1*, *PSMA1*, *PSMA2*, *PSMA3*, *PSMA5*, *PSMA6*, *PSMA7*, *PSMB1*, *PSMB3*, *PSMB5*, *PSMC1*, *PSMC2*, *PSMC3*, *PSMC4*, *PSMC5*, *PSMC6*, *PSMD1*, *PSMD11*, *PSMD13*, *PSMD14*, *PSMD2*, *PSMD3*, *PSMD4*, *PSMD6*, *PSME3*, *PUM2*, *ROCK2*, *SAMD4B*, *SERBP1*, ***SYNCRIP***, ***THRAP3***, *UBC*, *UPF1*, *XPO1*, *YTHDF1*, *YTHDF2*, *YTHDF3*, *YWHAB*, *YWHAZ*
Regulation of mRNA splicing via the spliceosome (GO:0048024)	7.6E^{-36}	***CELF1***, *CELF2*, *CELF4*, *CELF5*, *DAZAP1*, *DDX17*, ***DDX5***, ***FXR1***, ***FXR2,*** *HNRNPL*, ***HSPA8***, *IK*, *KHDRBS3*, ***MAGOH***, *NCBP1*, *NCL*, *NOVA1*, ***PRPF19***, *QKI*, *RBFOX1*, *RBFOX2*, *RBM17*, *RBM25*, *RBM39*, *RBM4*, *RBM5*, *RNPS1*, *SART3*, *SF1*, *SF3B4*, *SFSWAP*, *SMU1*, *SNRNP70*, ***SNW1***, *SRPK2*, ***SRSF1***, *SRSF2*, *SRSF3*, *SRSF4*, *SRSF7*, *SRSF9*, ***THRAP3***, *TRA2B*, *U2AF2*, *WTAP*, *YTHDC1*, *ZBTB7A*
Catalytic step two of the spliceosome (GO:0071013)	1.3E^{-22}	*CDC5L*, *CWC15*, *DDX23*, ***DDX5,*** *HNRNPA3*, ***HNRNPC***, *HNRNPF*, *HNRNPH1*, ***HNRNPM***, ***HNRNPR, MAGOH***, ***PABPC1,*** *PLRG1*, ***PRPF19***, *PRPF4B*, *PRPF6*, *PRPF8*, *RBM22*, *SART1*, *SF3A1*, ***SF3A2***, *SF3A3*, *SF3B1*, *SF3B2*, *SF3B3*, *SLU7*, *SNRNP200,SNRPD3*, ***SNW1,*** *SRRM1*, *SRRM2*, ***SRSF1***, ***SYNCRIP***, *XAB2*

FDR: false discovery rate; **bold** genes are represented two or more times in this table.
