# Supplementary material for: NeuroSCORE is a genome-wide omics-based model that identifies candidate disease genes of the central nervous system
Source: Sci Rep. 2022 Mar 31;12:5427. doi: 10.1038/s41598-022-08938-y (PMC8971396; doi:10.1038/s41598-022-08938-y)
Supplement: Supplementary file 4 — Supplementary Information 4. [file 41598_2022_8938_MOESM4_ESM.pdf]

**Supplementary Table S4: High Scoring Genes Associated with Non-CNS Phenotypes in OMIM NeuroSCORE Genes**

| NeuroSCORE | Genes |
|---|---|
| 5 | *ANK2, MYH9, WNK1* |
| 4 | *ACTN4, ADD1, ATP2B2, BAP1, BMPR2, CALM1, CHMP4B, CNBP, CTNNA1, CTNND1, CUL3, DCAF8, DYRK1B, FN1, FXR1, GANAB, GNAS, JAK1, MEF2A, MYO9B, PICALM, PRPF8, PSMA6, RASA1, SF3B1, SF3B4, SNRNP200, STAT1, STK11, TOP1* |
| 3 | *ACVR1B, ADCY1, ATG16L1, ATP1B1, ATP2C1, BCR, CALM2, CALM3, CFL2, COL6A1, COPA, CTSB, CYLD, DKC1, EGLN1, EHBP1, EWSR1, EXOC6B, FHL1, G6PD, GDF11, GPRASP2, HMGA1, HMGCR, HNRNPDL, IFNGR2, IKZF1, INF2, IRF2BP2, IRS2, KCNH2, KIF1B, KIF21A, LRP1, LTBP3, MAGED2, MAPK8IP1, MAPRE2, MECOM, MEN1, MFN2, MLH1, MORC2, MSN, NCSTN, NFKBIA, NLRP1, NPM1, NUMA1, P4HB, PABPN1, PAX5, PIP5K1C, PKD1, PLS3, PRCC, PRG4, PRKAG2, PRPF3, PRPF31, PRPF6, PTPRF, PTPRZ1, RAB7A, RAP1GDS1, RB1CC1, RELA, RIMS1, RPL11, RPL13, RPL15, RPL18, RPL5, RPS10, RPS15A, RPSA, SEPTIN9, SERPING1, SORT1, SPRY2, STAT3, STAT5B, SUMO1, TAB2, TCF7L2, TFE3, TNPO3, TPM3, TRAF3, TSG101, WARS1* |

OMIM database accessed July 31st, 2021
