# Supplementary material for: NeuroSCORE is a genome-wide omics-based model that identifies candidate disease genes of the central nervous system
Source: Sci Rep. 2022 Mar 31;12:5427. doi: 10.1038/s41598-022-08938-y (PMC8971396; doi:10.1038/s41598-022-08938-y)
Supplement: Supplementary file 5 — Supplementary Information 5. [file 41598_2022_8938_MOESM5_ESM.pdf]

Supplementary Table S5: Details for 31 Studies Used for the *De Novo* Metric in NeuroSCORE

Study PMID	Cohort Description	Affected Cohort Size
22365152	Family quartet with proband with severe epileptic encephalopathy.	1
23020937	51 participants from the German Mental Retardation Network.	51
23033978	100 patients with an IQ below 50 and their unaffected parents.	100
23086397	Three probands with Malignant migrating partial seizures of infancy.	3
23260136	“Ten MZ twin pairs concordant for autism spectrum disorder and their parents were obtained from the NIMH genetics initiative biorepository.”	20
23647072	Ten children with “sporadic epilepsy characterized by difficult-to-control seizures and some combination of developmental delay, epileptic encephalopathy, autistic features, cognitive impairment, or motor deficits.”	10
23911319	105 probands with schizophrenia	105
23934111	264 trios with either infantile spasms or Lennox-Gastaut syndrome collected through the Epilepsy Phenome/Genome Project.	264
24463507	“Parent proband trios (N=623), where the proband had a history of hospitalization for schizophrenia or schizoaffective disorder, were recruited from psychiatric hospitals in Bulgaria…617 probands (6 trios were excluded after QC)…”	617
24501278	Identical twins with ASD and seizures	2
24650168	Case report of singleton with ASD	1
24776741	“In this study we have sequenced the exome of 171 individuals representing 42 sporadic and 15 familial trios with schizophrenia or a related psychotic condition to identify additional risk mutations. All subjects from Ireland.”	57
25363760	Combines cases from sixteen affected cohorts: PAGES Swedish cases (25038753); Simons Simplex (22495306; 22495309; 22542183); Seaver ASD Assessment center, TASC, and two groups from UK 10k trios (Described in Supplementary Table 1).	3871
25666757	98 case-parent trios with probands affected with cerebral palsy.	98
25805808	43 sporadic cases affected with myelomeningocele or anencephaly and their unaffected parents.	43
26091878	“In our clinical research, we cast a net for genes associated with schizophrenia, sequencing 14 sporadic offspring-parent trios from the Jerusalem Perinatal Schizophrenia Study sample.”	14
26138355	10 probands with infantile spasms and their unaffected parents.	10
26194182	12 trios with probands diagnosed with early-onset Alzheimer disease	12

26352270	“WES was applied in search of *de novo* variants that might be causative of ASD in four family trios from Colombia.”	4
26362251	“We performed exome sequencing in full parent-child trios where the proband presents with typical Parkinson's disease to unequivocally identify de novo mutations…in 21 trios.”	21
26582266	“The cohort consisted of 30 patients with ASD (21 males and 9 females) and their parents. All participants are Japanese and were recruited from either outpatient or inpatient services at the Osaka University Hospital.”	30
26795593	254 patients with epilepsy diagnoses	254
27217147	“We used DNA samples from 79 trios with a bipolar disorder proband (56 with bipolar I disorder (BDI) and 23 with bipolar II disorder (BDII)) and unaffected parents.”	79
27334371	39 parent-patient trios recruited from the Genetic Diagnostics Unit at Uppsala University Hospital where the patients had intellectual disability in combination with epilepsy.	39
27479843	820 individuals with intellectual disability ranging from mild (IQ 50-70) to severe-profound (IQ<30).	820
27525107	“We selected 200 unrelated trio families from a cohort of Canadian ASD families, based on the fact that the index case (proband) was the only affected individual in the family at the time of proband’s diagnosis (simplex families).”	200
27626066	Case report of a child with childhood-onset schizophrenia.	1
28135719	“At 24 clinical genetics centers within the United Kingdom (UK) National Health Service and the Republic of Ireland, 4,293 patients with severe, undiagnosed developmental disorders and their parents…”	4,293
28263302	Multiple previously sequenced cohorts. “Modifying our previous approaches (Methods), we studied those 1,239 families (1,627 parents-child trios) for which child and parental WGS data were available…”	1,627
28472652	“We have completed WES of 325 Tourette disorder trios from the Tourette International Collaborative Genetics cohort and a replication sample of 186 trios from the Tourette Syndrome Association International Consortium on Genetics (511 total).”	511
28608572	“Exome analysis was conducted for sporadic Tourette syndrome cases: nine trio families and one quartet family with concordant twins were investigated.”	10
